# Effectiveness of a large language model for clinical information retrieval regarding shoulder arthroplasty

**DOI:** 10.1002/jeo2.70114

**Published:** 2024-12-17

**Authors:** Jacob F. Oeding, Amy Z. Lu, Michael Mazzucco, Michael C. Fu, David M. Dines, Russell F. Warren, Lawrence V. Gulotta, Joshua S. Dines, Kyle N. Kunze

**Affiliations:** ^1^ Department of Orthopaedics, Institute of Clinical Sciences, The Sahlgrenska Academy University of Gothenburg Gothenburg Sweden; ^2^ Weill Cornell Medical College New York New York USA; ^3^ Department of Orthopaedic Surgery Hospital for Special Surgery New York New York USA; ^4^ Sports Medicine and Shoulder Institute Hospital for Special Surgery New York New York USA

**Keywords:** ChatGPT, information retrieval, large language model, LLM, total shoulder arthroplasty

## Abstract

**Purpose:**

To determine the scope and accuracy of medical information provided by ChatGPT‐4 in response to clinical queries concerning total shoulder arthroplasty (TSA), and to compare these results to those of the Google search engine.

**Methods:**

A patient‐replicated query for ‘total shoulder replacement’ was performed using both Google Web Search (the most frequently used search engine worldwide) and ChatGPT‐4. The top 10 frequently asked questions (FAQs), answers, and associated sources were extracted. This search was performed again independently to identify the top 10 FAQs necessitating numerical responses such that the concordance of answers could be compared between Google and ChatGPT‐4. The clinical relevance and accuracy of the provided information were graded by two blinded orthopaedic shoulder surgeons.

**Results:**

Concerning FAQs with numeric responses, 8 out of 10 (80%) had identical answers or substantial overlap between ChatGPT‐4 and Google. Accuracy of information was not significantly different (*p* = 0.32). Google sources included 40% medical practices, 30% academic, 20% single‐surgeon practice, and 10% social media, while ChatGPT‐4 used 100% academic sources, representing a statistically significant difference (*p* = 0.001). Only 3 out of 10 (30%) FAQs with open‐ended answers were identical between ChatGPT‐4 and Google. The clinical relevance of FAQs was not significantly different (*p* = 0.18). Google sources for open‐ended questions included academic (60%), social media (20%), medical practice (10%) and single‐surgeon practice (10%), while 100% of sources for ChatGPT‐4 were academic, representing a statistically significant difference (*p* = 0.0025).

**Conclusion:**

ChatGPT‐4 provided trustworthy academic sources for medical information retrieval concerning TSA, while sources used by Google were heterogeneous. Accuracy and clinical relevance of information were not significantly different between ChatGPT‐4 and Google.

**Level of Evidence:**

Level IV cross‐sectional.

**Abbreviations**
AI: artificial intelligence
FAQ: frequently asked question
GB: gigabyte
GPT: Generative Pre‐trained Transformer
LLM: large language model
TSA: total shoulder arthroplasty

## INTRODUCTION

Recently deployed large language models (LLMs) have demonstrated far‐reaching applications and impressive capabilities, allowing for the completion of several natural language tasks such as question answering, information retrieval, summarization, and imaging interpretation. It is plausible that the applications of LLMs in clinical medicine are poised for expediting infrastructural administrative processes and unlocking novel methods for task completion within health care and many commercial and private sectors [1, 5]. Chat‐Generative Pre‐trained Transformer (GPT), a consumer‐facing LLM and AI chatbot, has garnered a considerable amount of interest for the successful performance of such tasks and dynamic ability to adapt and learn. Indeed, ChatGPT‐4 has demonstrated the ability to pass several professional exams [9, 12], provide accurate knowledge concerning the subject matter [4], and coordinate complex tasks through integrating multiple resources [3], all of which have culminated in substantial investments with planned integration into commercial and medical software.

The unsupervised functionality of ChatGPT‐4 is potentially advantageous for patients seeking to gain deeper insight into specific musculoskeletal conditions, as ChatGPT‐4 can use auxiliary data without task‐specific training. Therefore, this allows patients to use the platform to rapidly obtain answers to an inquiry using non‐specific inputs [15, 17]. Indeed, prior literature concerning total hip and knee arthroplasty has demonstrated that ChatGPT‐4 can provide credible information for certain open‐ended and discrete questions [7]; however, verification of this information and corroboration with health care professionals in the early stages is necessary. The annual number of total shoulder arthroplasty (TSA) procedures is growing at a rate greater than that of total hip and knee arthroplasty [8, 16], and therefore the number of patient queries pertaining to the evaluation, management and recovery after TSA may anecdotally be expected to increase. The capabilities of ChatGPT‐4 may also reduce the burden of responding to patient inquiries concerning their current diagnoses or post‐operative instructions that are regularly imposed on surgeons and ancillary staff. Therefore, with the rapid dissemination and easy accessibility of ChatGPT‐4 to patients, it is important to determine whether the information being provided by this platform is credible.

Given the potential implications of utilizing ChatGPT‐4 as a source of medical information concerning musculoskeletal conditions, it is imperative to assess the potential utility of ChatGPT‐4 such that it can be advocated for or cautioned against for patient use. This technology may also have purported benefits for shoulder and elbow surgeons, wherein specific post‐operative rehabilitation and peri‐operative care guidelines are integrated into an LLM and available for patients under their care to query as needed.

The purpose of this study was to determine the scope and accuracy of medical information provided by ChatGPT‐4 in response to clinical queries concerning TSA, and to compare these results to those of the Google search engine. The authors hypothesized that medical information retrieved by ChatGPT‐4 would be accurate as assessed by expert graders with domain expertise and that the sources used to provide information would be trustworthy.

## MATERIALS AND METHODS

### Language model and architecture

In the current study, the LLM Chat Generalized Pre‐Trained Transformer–4 (ChatGPT‐4; Release: GPT‐4 default mode non‐plugin enhanced), the most recent version of the pre‐trained transformer Chatbot created by OpenAI and first released on 14 March 2023, was used.

ChatGPT‐4 is a state‐of‐the‐art LLM that functions through advanced natural language processing to generate responses based on word association and attention. ChatGPT‐4 was largely trained using text databases from the internet, consisting of over 570 GB of data and resulting in 300 billion words being fed into the system to develop the Chatbot [14]. Through supervised and reinforcement training techniques, the transformer network of ChatGPT‐4 has been developed such that it can provide sophisticated and insightful responses concerning a diverse variety of human‐generated commands and queries [14]. Any query history was deleted prior to the initiation of queries in order to mitigate the risk of bias from memory.

### Search strategy, information retrieval and patient query replication process

Both Google Web Search and ChatGPT‐4 were utilized to perform independent searches. The Google Web Search was performed utilizing a clean‐installed Google Chrome Browser on 20 November 2023. A clean‐installed browser was utilized in order to minimize contributions of individualized search algorithms and bias potentially present in sponsored advertisements, cookies, cache and browsing history. Google was chosen as the default search engine for comparison as it is the most widely used search engine worldwide and the only search engine that generates frequently asked questions (FAQs) when prompted with a query, therefore allowing comparisons between Google and ChatGPT‐4 through a systematic approach [13].

Beginning first with Google, the search term ‘total shoulder replacement’ was entered. The first 10 FAQs, in conjunction with the associated sources, were recorded. All questions pertaining to total shoulder replacement, including those with alternative terms such as ‘total shoulder arthroplasty’, ‘shoulder arthroplasty’ and ‘shoulder replacement’ were considered. FAQs were excluded if they were not relevant to TSA or if they represented a duplicate question. The approach focusing on FAQs was strategically chosen as it (1) inherently represents the questions generating the most interest and importance for those performing searches on the topic, (2) allowed for a systematic method of question generation and recording without bias introduced by the authors, and (3) provided a reproducible method of question generation to compare between Google and ChatGPT‐4. The same query was then input into ChatGPT‐4 in order to determine the top 10 FAQs, where the answers and their associated sources were also recorded for comparison. ChatGPT‐4 was manually prompted to provide the source of its answers as it does not automatically provide this information. This process was again repeated with a clean history for both Google and ChatGPT‐4 to identify the top 10 FAQs concerning numeric, or discrete, answers concerning TSA.

### Expert content accuracy assessment

Answers were assessed for accuracy based on the clinical judgement of two fellowship‐trained sports medicine and shoulder surgeons. The criteria for selecting these experts included completion of a sports medicine and/or shoulder and elbow fellowship and a practice with primary specialization in shoulder surgery. These reviewers were blind to whether the answer was from Google or ChatGPT‐4, as well as the search engine's source of information. Each answer was rated on a 5‐point Likert scale as either completely incorrect (0 points), more incorrect than correct (1 point), approximately equal correct and incorrect (2 points), more correct than incorrect (3 points) or completely correct (4 points), which has been consistently applied and deemed as a valid scale in prior literature concerning ChatGPT [6, 10]. Furthermore, each blinded rater assessed the clinical relevance of provided FAQs on a 5‐point Likert scale as completely irrelevant (0 points), more irrelevant than relevant (1 point), approximately equal relevant and irrelevant (2 points), and more relevant than irrelevant (3 points), and completely relevant (4 points). The clinical relevance of FAQs with numeric answers was not compared since FAQs did not differ between the search engines and the goal was to compare objective numeric responses for the same FAQs.

### Statistical analysis

Each FAQ was categorized into question topics described as (1) Fact, (2) Policy or (3) Value as according to the Rothwell Classification in accordance with prior literature (Table 1) [11]. Questions were also subcategorized within these three distinctions into ten topics that are clinically relevant to TSA. Websites were also categorized into predefined groups based on prior literature (Table 1) [7]. Academic sources were the only sources considered trustworthy as they provided evidence‐based information. All categorizations were performed by two independent raters (M.M. and K.N.K.) and confirmed by a third party if discrepancies arose. Categorizations were subsequently compared between Google and ChatGPT‐4. The number of academic sources used by Google and ChatGPT‐4 were compared using Chi‐squared and Fisher's exact test depending on cell event frequency where appropriate. All statistical analyses were performed with Microsoft Excel (Microsoft) and GraphPad Prism Version 9.5.1 (GraphPad Software), with statistical significance being defined by using a *p* < 0.05 threshold in all circumstances.

**Table 1:** Rothwell classification for question types for internet content and website classifications.

**Rothwell classification**	
**Fact**	**Ask whether something is true, and to what extent**
Specific activities	Ability to perform a specific activity or action
Timeline of recovery	Length of time for recovery milestones
Technical details	Surgical procedure and anaesthesia, including specifics about implants
Restrictions	Restrictions to activity or lifestyle during recovery
Cost	Cost of surgery and/or rehabilitation
**Policy**	**Ask whether a certain course of action should be taken to solve a problem**
Indications/management	Surgical indications and alternatives, post‐operative management, timing of surgery
Risks/complications	Risks/complications before, during or after THA/TJA, including rehabilitation period
**Value**	**Ask for evaluation of an idea, object or event**
Pain	Related to the timing, severity and management of pain
Longevity	Longevity of TSA
Evaluation of surgery	Successfulness, seriousness, or invasiveness of TSA
**Website categorization**	
Commercial	Organizations that provide public health information, including medical device/manufacturing/pharmaceutical companies and news outlets
Academic	Universities, academic medical centres or academic societies
Medical practice	Local hospitals or medical groups without clear academic affiliation
Single surgeon practice	Personal websites maintained by individual surgeons
Government	Websites maintained by a national government
Social media	Blog, internet forms, support groups and non‐medical organizations designed for information and video sharing

Abbreviations: THA, total hip arthroplasty; TJA, total joint arthroplasty; TSA, total shoulder arthroplasty.

## RESULTS

### Replicated patient queries: Open‐ended FAQs

The top 10 FAQS concerning TSA and their subcategorizations on Google and ChatGPT‐4 are outlined in Table 2. The answers to these FAQs by ChatGPT‐4 and Google can be found in Appendix S1. Actual questions overlap between FAQs were observed in only 3 out of 10 (30%) questions elicited from the query. The most common FAQ category according to the Rothwell Classification was Fact (7 out of 10, 70%) for Google, while the most common for ChatGPT‐4 were Fact (4 out of 10, 40%) and Policy (4 out of 10, 40%).

**Table 2:** Top 10 Internet FAQs for TSA per Google and ChatGPT‐4.

Google	Mean relevance	ChatGPT‐4	Mean relevance
1. What is total shoulder arthroplasty? **(Technical Details)**	4	1. What is total shoulder arthroplasty (TSA)? **(Technical Details)**	4
2. Is total shoulder arthroplasty the same as shoulder replacement? **(Technical Details)**	4	2. How is total shoulder arthroplasty performed? **(Technical Details)**	3.5
3. How long does it take to recover from total shoulder replacement? **(Timeline of Recovery)**	4	3. Who is a candidate for total shoulder arthroplasty? **(Indications/Management)**	4
4. How painful is total shoulder replacement? **(Pain)**	4	4. What are the risks and complications associated with total shoulder arthroplasty? **(Risks/Complications)**	4
5. Is shoulder arthroplasty a major surgery? **(Evaluation of Surgery)**	3	5. How long does it take to recover from total shoulder arthroplasty? **(Timeline of Recovery)**	4
6. What is the risk or downside of a shoulder replacement? **(Risks/Complications)**	4	6. What is the difference between total shoulder arthroplasty and reverse total shoulder arthroplasty? **(Technical Details)**	4
7. What is the average age for shoulder replacement? **(Indications/Management)**	4	7. How successful is total shoulder arthroplasty in terms of pain relief and improved function? **(Evaluation of Surgery)**	4
8. How long do you have to sleep in a recliner after shoulder surgery? **(Restrictions)**	3.5	8. What is the typical lifespan of a total shoulder arthroplasty implant? **(Evaluation of Surgery)**	4
9. Are there permanent restrictions after shoulder replacement? **(Restrictions)**	4	9. Are there any alternatives to total shoulder arthroplasty? **(Indications/Management)**	4
10. Do you still have a rotator cuff after shoulder replacement? **(Technical Details)**	3	10. What types of physical therapy or rehabilitation are recommended after total shoulder arthroplasty? **(Indications/Management)**	4

*Note*: Highlighted green cells indicated FAQs considered identical. Parentheses indicate Rothwell Classification.

**Figure 1:**
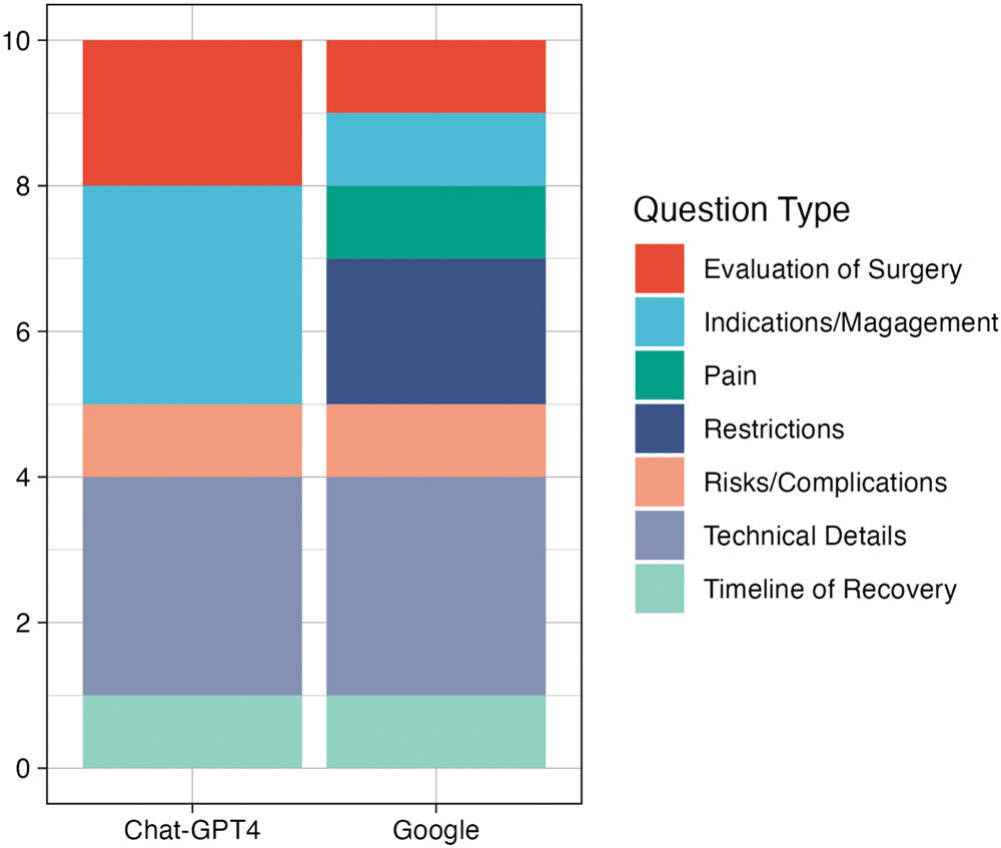
Comparison of question categorizations for general frequently asked questions between ChatGPT‐4 and Google.

The most common subcategory by topic (Figure 1) was technical details (3 out of 10, 30%) for Google, while the most common FAQs for ChatGPT‐4 concerned indications/management (3 out of 10, 30%) and technical details (3 out of 10, 30%). In terms of sources, Google utilized predominately academic (6 out of 10, 60%) and also included social media (20%), medical practice (10%) and single‐surgeon practice (10%), while 100% of sources for ChatGPT‐4 were academic. The frequency of utilization of academic sources was significantly greater when ChatGPT‐4 was used to provide answers compared with Google (*p* = 0.025).

Blinded assessment of clinical relevance of these FAQs resulted in a mean (±standard deviation) accuracy validity score of 3.95±0.16 for ChatGPT‐4 versus 3.75 ± 0.42 for Google, which was not a statistically significant difference (*p* = 0.18). Furthermore, the proportion of completely relevant answers was not significantly different between the two platforms (ChatGPT: 9 vs.Google: *p* = 7 and *p* = 0.58).

### Replicated patient queries: Discrete‐numeric FAQs

**Figure 2:**
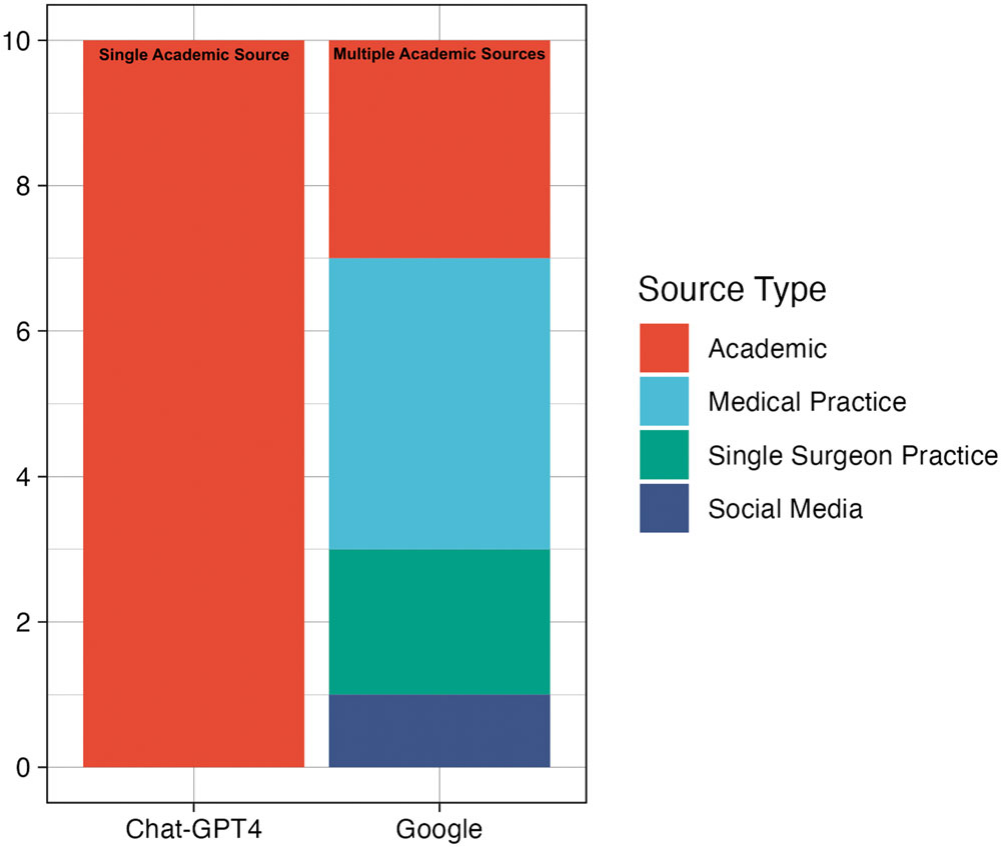
Comparison of categorization of internet sources used to answer frequently asked questions with numeric‐discrete answers.

The top 10 numerical FAQS concerning TSA and their subcategorizations on Google and ChatGPT‐4 are outlined in Table 3. In these queries, 8 out of 10 (80%) FAQs had substantial overlap or identical answers between the search engines. Sources for Google included 40% medical practices, 30% academic, 20% single‐surgeon practice and 10% social media, while ChatGPT‐4 used 100% academic sources. ChatGPT‐4 provided answers using a significantly greater proportion of academic resources compared with Google (*p* = 0.001; Figure 2).

**Table 3:** Top 10 Internet numeric‐based FAQs for TSA and comparison of answers between Google and ChatGPT‐4.

Google numerical FAQs	Google answer	Mean accuracy	ChatGPT‐4 answer	Mean accuracy
1. What is the average age for shoulder replacement?	60–80 years old **(Medical Practice)**	4	Over 50 years old **(Academic)**	4
2. How long do you have to sleep in a recliner after shoulder surgery?	4–6 weeks **(Single Surgeon Practice)**	1	4–6 weeks **(Academic)**	1
3. How long does it take to recover from total shoulder replacement?	At least 6 months **(Academic)**	3	Within 3–6 months (**Academic)**	4
4. How many hours is a total shoulder replacement?	About 3 h (**Academic)**	2	Up to 3 h **(Academic)**	3
5. How long do you wear a sling after total shoulder replacement surgery?	4–6 weeks post‐operatively **(Single Surgeon Practice)**	4	4–6 weeks (**Academic)**	4
6. What percentage of shoulder replacements are successful?	90% **(Medical Practice)**	2.5	90%–95% **(Academic)**	4
7. How long is the hospital stay for shoulder replacement surgery?	1–2 nights **(Academic)**	4	1–3 days **(Academic)**	4
8. How big is the incision for total shoulder replacement?	6 in. **(Social Media)**	4	4–6 in. **(Academic)**	4
9. What is the most painful day after surgery?	Days 2 and 3 **(Medical Practice)**	3	First few days after surgery **(Academic)**	3
10. How painful is shoulder surgery on a scale from 1 to 10?	6 out of 10 **(Medical Practice)**	2.5	Pain levels after shoulder surgery can vary. However, with appropriate pain management, most patients can expect their pain to be manageable during the recovery process. **(Academic)**	3.5

*Note*: Highlighted green cells indicated responses considered identical, yellow cells indicated responses with substantial overlap and red cells indicated responses with no overlap. Parentheses indicate the website source category.

Abbreviation: TSA, total shoulder arthroplasty.

Blinded assessment by the clinical experts resulted in a mean (±standard deviation) accuracy validity score of 3.45 ± 0.96 for ChatGPT‐4 versus 3.0 ± 1.0 for Google, which was not a statistically significant difference (*p* = 0.32). Furthermore, the proportion of completely accurate answers was not significantly different between the two platforms (ChatGPT: 6 vs. Google: 4; *p* = 0.65).

## DISCUSSION

The main findings of the current study are as follows: (1) ChatGPT‐4 can provide a comprehensive range of clinically relevant questions and answers; (2) a large proportion of overlap concerning FAQs with numerical answers was observed between ChatGPT‐4 and Google; (3) all derived information to generate questions and answers were from trustworthy academic sources for ChatGPT‐4; and (4) the accuracy and clinical relevance of information was not significantly different between ChatGPT‐4 and Google. Taken together, the findings from the current study suggest information on TSA that patients obtain from ChatGPT‐4 is from high quality sources and unlikely to be worse than when obtained from Google.

The current study systematically analyzed the accuracy and clinical relevance of FAQs generated by ChatGPT‐4 and compared them to the most frequently used search engine worldwide. In terms of replicating FAQs, Chat‐GPT generally demonstrated discordance from Google search, with only 30% of FAQs being identical or having substantial overlap. However, this discordance suggests that ChatGPT‐4 could be implemented as an augment for information that may not be readily accessible through Google. Furthermore, this study provides evidence that ChatGPT‐4 possesses the capability to appropriately interpret patient questions and perform medical information retrieval utilizing a significantly greater proportion of trustworthy academic sources when compared to Google. Future iterations and refinement to the information retrieval process of ChatGPT‐4, as well as rigorous tests of safety and accuracy of the breadth of information that ChatGPT‐4 can provide, may allow it to be deployed into clinical settings to optimize the efficiency of patient outreach and clinical workflow.

The selection process for why ChatGPT‐4 uses a high proportion of trustworthy, academic sources is unclear as it is not disclosed information and is related to the inherent training architecture of the model; however, the statistically significant difference in favour of ChatGPT‐4 for providing answers using academic sources may be a representation of the sources used to train the model and the time period during which this was accomplished. This is in opposition to Google, which has access to resources over a larger time period and is not confined by the time period during which Google was created. A high proportion of academic sources in generating medical answers by ChatGPT has been observed in prior literature [7]. This suggests several potential benefits and disadvantages in the way in which ChatGPT‐4 currently functions to provide medical information. An advantage of outputs being a reflection of the model training is the ability for developers to create novel retrieval‐augmented language models that are trained specifically on medical‐specific information databases. For example, by using predetermined online domains for information retrieval, or by creating a database containing specific types of medical knowledge relevant to a condition (such as indications, surgical considerations, and patient recovery knowledge concerning shoulder arthroplasty), confidence in the information being provided could be increased. Furthermore, the consistency of using this single source may prevent patients from being presented with conflicting information procured from various sources with differing answers, thereby preventing unnecessary confusion from attempting to integrate information from several resources. This may also decrease the burden on providers to rectify these discrepancies. However, the consistent use of a single source also may result in bias in the training data that the LLM has been trained on, which could be subsequently propagated. These LLMs could also be subject to adversarial prompting and errors of input, further biasing information. This could become legally and ethically problematic should patients be presented with misleading information used to make decisions on treatment.

Fewer disparities were observed when comparing questions and answers between ChatGPT‐4 and Google when concerning discrete numeric queries. Indeed, 80% of these answers provided identical numeric responses or a range of numbers with substantial overlap. This finding is clinically important as it demonstrates that ChatGPT‐4 is credible and has the potential to be used by patients as an accurate source of information. However, regardless of the response, it is essential that patients confirm answers with a specialized healthcare provider in order to avoid patient‐expectation mismatches and delays in care. When providing numeric responses, ChatGPT‐4 used academic resources in 100% of cases, whereas Google used 40% medical practices, 30% academic, 20% single‐surgeon practices and 10% social media, suggesting heterogeneity in the resources provided to patients. This suggests that ChatGPT‐4 also uses sources with presumably more reliable evidence in comparison to Google when considering this subset of questions. Future studies are warranted to determine the credibility of ChatGPT‐4 for providing patient information on a broader variety of topics. With increased validity and training, it is possible that ChatGPT may be incorporated as a tool within provider platforms and provide automated information, such as sending real‐time responses for providers to review, edit and send to patients. Furthermore, ChatGPT could be implemented to route patients to an appropriate provider given their questions and symptoms (i.e., someone with cervical radiculopathy is routed to see an orthopaedic spine surgeon as opposed to a shoulder and elbow surgeon).

ChatGPT‐4 is a rapidly evolving tool with vast applications and the potential to improve patient care and decrease provider and administrative burdens. For example, should ChatGPT‐4 be allowed autonomously function as a first‐line provider for medical professionals to answer patient questions on their behalf, administrative burden on advanced care practitioners and clinicians may be decreased and patient satisfaction enhanced. Previous studies have demonstrated that ChatGPT‐4 can potentially function in this manner and be more empathetic [2]; however, in its current form, ChatGPT‐4 should be utilized as an adjunct at best and remain only a potential resource for patients. Prior studies have demonstrated that the quality and reliability of popular internet resources for patients may be limited and regulations that implement a vetting and peer‐review process are necessary. For ChatGPT‐4, iterative model training and validation are essential prior to the ability to depend on the model alone for credible medical information. It is commendable and reassuring that ChatGPT‐4 used 100% academic resources to provide responses to queries concerning TSA; however, further research into the reproducibility and validity of this use is warranted.

Finally, it is important to discuss future directions for this work given the rapid pace at which LLMs are evolving. These include demographic considerations as well as patient‐reported outcomes. Future work may entail examining how different demographic groups interact with ChatGPT‐4 and Google. Factors like age and education level may influence how users interpret or trust responses, which could be valuable to explore in a follow‐up study. In addition, incorporating patient feedback on the usefulness, clarity, and trustworthiness of ChatGPT‐4's answers could provide valuable insight. This would add a patient‐centred perspective and validate the model's utility in real‐world settings, which could also be evaluated in a follow‐up study.

### Limitations

Several limitations are important to consider in the context of the current study results. First, patients may use multiple resources to perform inquiries and procure information, which is not captured in the current study. The comparison with only Google limits the scope of the results. Including other popular platforms like YouTube could give a better understanding of how ChatGPT‐4 performs compared to other used sources. Second, given the cross‐sectional nature of this study design, potential confounding factors that may influence internet use, such as socioeconomic and demographic factors, were not controlled for. Third, the current study assessed a limited number of questions to determine the degree to which ChatGPT‐4 could replicate a Google search concerning TSA, which may restrict the generalizability of its findings and expand the range of queries, both in volume and scope, could provide a more comprehensive evaluation. It is also important to note that the searches were performed in a single geographic location, and any potential effect on search results conferred by geography/location cannot be controlled should they exist. Finally, given the rapid pace at which LLMs are evolving and new models are being released, it is possible that a new version of ChatGPT may have been released since the publication of this study. Thus, the performance of newer models may differ from that of ChatGPT‐4, which was the version used in the present study. However, it is likely that as LLMs continue to advance, their performance will only further improve, and they may be refined and tuned to ensure the provision of trustworthy information from reliable sources.

## CONCLUSION

ChatGPT‐4 provided trustworthy academic sources for medical information retrieval concerning TSA, while sources used by Google were heterogeneous. The accuracy and clinical relevance of information were not significantly different between ChatGPT‐4 and Google.

## AUTHOR CONTRIBUTIONS

**Kyle N. Kunze**: Conceptualization; writing of initial manuscript; supervision; manuscript revision. **Amy Z. Lu** and **Michael Mazzucco**: Data procurement and analysis. **Jacob F. Oeding**, Michael C. Fu, **David M. Dines**, **Russell F. Warren**, **Lawrence V. Gulotta** and **Joshua S. Dines**: Manuscript revision.

## CONFLICT OF INTEREST STATEMENT

The authors declare no conflicts of interest.

## ETHICS STATEMENT

This study was exempt from institutional review board approval at the Hospital for Special Surgery.

## Supporting information

Supporting information.

## Data Availability

The data that support the findings of this study are available from the corresponding author upon reasonable request.
