# Supplementary material for: Effectiveness of a large language model for clinical information retrieval regarding shoulder arthroplasty
Source: J Exp Orthop. 2024 Dec 17;11(4):e70114. doi: 10.1002/jeo2.70114 (PMC11649951; doi:10.1002/jeo2.70114)
Supplement: Supplementary file 1 — Supporting information. [file JEO2-11-e70114-s001.docx]

**Appendix I**. Answers to top ten FAQS provided by ChatGPT and Google.

| **FAQ** | **Answer** | **Source** |
| --- | --- | --- |
| *ChatGPT* | | |
| 1. What is total shoulder arthroplasty (TSA)? | Total shoulder arthroplasty (TSA) is a surgical procedure that replaces the damaged or arthritic glenohumeral joint with artificial components, called prostheses. The procedure is performed to relieve pain and improve function in patients with shoulder arthritis or other shoulder joint disorders. | https://orthoinfo.aaos.org/  en/treatment/shoulder-  joint-replacement/ |
| 2. How is total shoulder arthroplasty performed? | Total shoulder arthroplasty is performed under general anesthesia or regional anesthesia. The surgeon makes an incision in the front of the shoulder, exposes the joint, removes the damaged bone and cartilage, and implants the prosthetic components. The humeral head is replaced with a metal ball, and the glenoid (socket) is replaced with a plastic component. | https://orthoinfo.aaos.org/  en/treatment/shoulder-  joint-replacement/ |
| 3. Who is a candidate for total shoulder arthroplasty? | Candidates for total shoulder arthroplasty typically have shoulder joint pain and dysfunction that has not responded to conservative treatments such as medications, physical therapy, or injections. Common indications include osteoarthritis, rheumatoid arthritis, post-traumatic arthritis, rotator cuff tear arthropathy, avascular necrosis, and certain severe fractures. | https://orthoinfo.aaos.org/  en/treatment/shoulder-  joint-replacement/ |
| 4. What are the risks and complications associated with total shoulder arthroplasty? | Risks and complications of total shoulder arthroplasty include infection, blood clots, anesthesia complications, nerve injury, fracture, dislocation, implant loosening, wear or failure, and the need for additional surgery. | https://orthoinfo.aaos.org/  en/treatment/shoulder-  joint-replacement/ |
| 5. How long does it take to recover from total shoulder arthroplasty? | Recovery time varies depending on the individual and the complexity of the surgery. Most patients can expect to regain functional use of their shoulder within 3 to 6 months after surgery, with continued improvement up to one year. | https://orthoinfo.aaos.org/  en/recovery/shoulder-  joint-replacement-recovery/ |
| 6. What is the difference between total shoulder arthroplasty and reverse total shoulder arthroplasty? | In total shoulder arthroplasty, the prosthetic components mimic the natural anatomy of the shoulder, with a metal ball replacing the humeral head and a plastic socket replacing the glenoid. In reverse total shoulder arthroplasty, the positions of the ball and socket components are switched: the ball is implanted on the glenoid side, and the socket is placed on the humerus. Reverse total shoulder arthroplasty is typically indicated when the rotator cuff is severely damaged or irreparable. | https://orthoinfo.aaos.org/  en/treatment/reverse-  total-shoulder-replacement/ |
| 7. How successful is total shoulder arthroplasty in terms of pain relief and improved function? | Total shoulder arthroplasty is generally successful in providing pain relief and improving function. Studies report that approximately 90% to 95% of patients experience significant pain relief and functional improvement after surgery. | https://orthoinfo.aaos.org/  en/treatment/shoulder-  joint-replacement/ |
| 8. What is the typical lifespan of a total shoulder arthroplasty implant? | The lifespan of a total shoulder arthroplasty implant varies depending on several factors, including the patient's age, activity level, and the specific implant used. Most modern implants have a lifespan of 10-20 years or more, but individual results may vary. | https://orthoinfo.aaos.org/  en/treatment/shoulder-  joint-replacement/ |
| 9. Are there any alternatives to total shoulder arthroplasty? | Alternatives to total shoulder arthroplasty may include non-surgical treatments such as medications, physical therapy, and injections for pain management. Surgical alternatives may include arthroscopic debridement, partial shoulder replacement (hemiarthroplasty), or joint resurfacing. | https://orthoinfo.aaos.org/  en/treatment/shoulder-  joint-replacement/ |
| 10. What types of physical therapy or rehabilitation are recommended after total shoulder arthroplasty? | Rehabilitation after total shoulder arthroplasty typically begins within the first day or two following surgery. Early-phase rehabilitation may include passive range-of-motion exercises, pain management, and the use of a sling for support. As the patient progresses, active range-of-motion exercises and strengthening exercises for the rotator cuff and scapular stabilizers are introduced. The specific protocol and timeline for rehabilitation may vary depending on the surgeon's recommendations and the patient's individual needs. | https://orthoinfo.aaos.org/  en/recovery/shoulder-  joint-replacement-recovery |
| *Google Web Search* | |  |
| 1. What is total shoulder arthroplasty? | Total shoulder arthroplasty is a joint replacement surgery for a variety of painful shoulder joint conditions. The ball and socket portion of your shoulder will be replaced with metal and plastic parts that are similar in shape and size to your own shoulder anatomy. | <https://www.ortho.wustl.edu/content/>  Patient-Care/3399/Services/Shoulder-  Elbow/Overview/Total-Shoulder-  Arthroplasty-/What-is-total-shoulder-arthroplasty.aspx#:~:text=Total%  20shoulder%20arthroplasty  %20is%20a,to%20your%20own  %20shoulder%20anatomy. |
| 2. Is total shoulder arthroplasty the same as shoulder replacement? | Shoulder replacement removes damaged areas of bone and replaces them with parts made of metal and plastic (implants). This surgery is called shoulder arthroplasty (ARTH-row-plas-tee). The shoulder is a ball-and-socket joint. | <https://www.mayoclinic.org/tests>  -procedures/shoulder-replacement/  about/pac-20519121#:~:text=Shoulder%  20replacement  %20removes%20damaged%20a  reas,ball%2Dand%2Dsocket%20joint. |
| 3. How long does it take to recover from total shoulder replacement? | Depending on your job, you may be able to go back to work as early as 2 to 3 weeks after surgery, as long as you avoid certain arm movements, such as lifting. It takes at least 6 months to return to full activity. | <https://myhealth.alberta.ca/Health/>  aftercareinformation/pages/  conditions.aspx?hwid=abo1301#  :~:text=Depending%20on%20your  %20job%2C%20you,to%20return%  20to%20full%20activity. |
| 4. How painful is total shoulder replacement? | You can expect some pain after shoulder replacement or any major surgery. Your pain will decrease as you heal. Most people report only trace to mild pain after they have fully recovered and rehabilitated from shoulder replacement surgery. | <https://www.choosept.com/health-tips>  /4-things-to-expect-shoulder-replacement-surgery#:~:text=You%20can%20expect%2  0some%20pain,rehabilitated%20from%2  0shoulder%20replacement%20surgery. |
| 5. Is shoulder arthroplasty a major surgery? | Total shoulder arthroplasty is a major surgical procedure that involves cutting of skin, tendons and bone. The pain from this surgery is managed by the anesthetic and by pain medications. | <https://orthop.washington.edu/>  patient-care/articles/shoulder/  total-shoulder-replacement-  arthroplasty-for-shoulder-arthritis.  html#:~:text=Total%  20shoulder%20arthroplasty%20is  %20a,are%20often%20  given%20by%20injection. |
| 6. What is the downside of a shoulder replacement? | What are the disadvantages of shoulder replacement surgery? Complications are relatively rare, occurring in 3-8% of patients, and include infection, fracture, instability, loosening of the prosthesis, continued pain and weakness, tearing of the rotator cuff and the need for revision surgery. | <https://www.bannerhealth.com/>  healthcareblog/better-me/are-you-  considering-shoulder-replacement-surgery#  :~:text=What%20are%20the%  20disadvantages%20of,the%20need%  20for%20revision%20surgery. |
| 7. What is the average age for shoulder replacement? | The typical age group for a shoulder replacement patient is 60-80 years old. I have performed shoulder replacements on patients as old as 88 and as young as the mid-40s. | <https://www.orthohealth.com/>  shoulder-replacement-surgery-  faq-with-dr-timothy-pater/#:~:text=The  %20typica  l%20age%20group%20for,young  %20as%20the%20mid%2D40s. |
| 8. How long do you have to sleep in a recliner after shoulder surgery? | Sleeping on an incline for 4-6 weeks after surgery is best. For the first week or two, a recliner may be the most comfortable option. Purchasing a 45 degree wedge from a medical supply store can also provide a stable base to prop yourself up in the bed. Wear your sling. | <https://www.kevinkrusemd.com/>  tips-for-sleeping-after-shoulder-  surgery/#:  ~:text=Sleeping%20on  %20an%20incline%20for,Wear  %20your%20sling. |
| 9. Are there permanent restrictions after shoulder replacement? | The full recovery from a shoulder replacement typically ranges from four to six months. That being said, some people's recoveries can be longer and sometimes shoulder replacement surgery can leave patients facing lifelong restrictions. | https://www.disability-benefits-help.org  /disabling-conditions/shoulder-replacement  #:~:text=Permanent%  20Restrictions%20After%20Shoulder%  20Replacement&text=The%20full  %20recovery  %20from%20a,leave%20patients%20facing  %20lifelong%20restrictions. |
| 10. Do you still have a rotator cuff after shoulder replacement? | The group of muscles and tendons that surround the shoulder joint (the rotator cuff) occasionally wear out after a partial or total anatomic shoulder replacement. | https://www.mayoclinic.org/tests-  procedures/shoulder-replacement/about  /pac-20519121#:~:text=The%20group%20of%  20muscles%20and,or%20total%  20anatomic%  20shoulder%20replacement. |
